# Modelling optimum use of attractive toxic sugar bait stations for effective malaria vector control in Africa

**DOI:** 10.1186/s12936-015-1012-9

**Published:** 2015-12-08

**Authors:** Lin Zhu, John M. Marshall, Whitney A. Qualls, Yosef Schlein, John W. McManus, Kris L. Arheart, WayWay M. Hlaing, Sekou F. Traore, Seydou Doumbia, Günter C. Müller, John C. Beier

**Affiliations:** Department of Public Health Sciences, Miller School of Medicine, University of Miami, Miami, FL USA; Divisions of Biostatistics and Epidemiology, School of Public Health, University of California, Berkeley, CA USA; Department of Microbiology and Molecular Genetics, Faculty of Medicine, IMRIC, Kuvin Centre for the Study of Infectious and Tropical Diseases, Hebrew University, Jerusalem, Israel; Department of Marine Biology and Ecology, Rosenstiel School of Marine and Atmospheric Science, University of Miami, Miami, FL USA; Malaria Research and Training Center, Faculty of Medicine, Pharmacy and Odonto-Stomatology, University of Bamako, BP 1805 Bamako, Mali

**Keywords:** Malaria, *Anopheles gambiae*, Attractive toxic sugar bait, ATSB, EIR, Individual-based model, Agent-based model

## Abstract

**Background:**

The development of insecticide resistance and the increased outdoor-biting behaviour of malaria vectors reduce the efficiency of indoor vector control methods. Attractive toxic sugar baits (ATSBs), a method targeting the sugar-feeding behaviours of vectors both indoors and outdoors, is a promising supplement to indoor tools. The number and configuration of these ATSB stations needed for malaria control in a community needs to be determined.

**Methods:**

A hypothetical village, typical of those in sub-Saharan Africa, 600 × 600 m, consisting of houses, humans and essential resource requirements of *Anopheles gambiae* (sugar sources, outdoor resting sites, larval habitats) was simulated in a spatial individual-based model. Resource-rich and resource-poor environments were simulated separately. Eight types of configurations and different densities of ATSB stations were tested. *Anopheles gambiae* population size, human biting rate (HBR) and entomological inoculation rates (EIR) were compared between different ATSB configurations and densities. Each simulated scenario was run 50 times.

**Results:**

Compared to the outcomes not altered by ATSB treatment in the control scenario, in resource-rich and resource-poor environments, respectively, the optimum ATSB treatment reduced female abundance by 98.22 and 91.80 %, reduced HBR by 99.52 and 98.15 %, and reduced EIR by 99.99 and 100 %. In resource-rich environments, n × n grid design, stations at sugar sources, resting sites, larval habitats, and random locations worked better in reducing vector population and HBRs than other configurations (*P* < 0.0001). However, there was no significant difference of EIR reductions between all ATSB configurations (*P* > 0.05). In resource-poor environments, there was no significant difference of female abundances, HBRs and EIRs between all ATSB configurations (*P* > 0.05). The optimum number of ATSB stations was about 25 for resource-rich environments and nine for resource-poor environments.

**Conclusions:**

ATSB treatment reduced *An. gambiae* population substantially and reduced EIR to near zero regardless of environmental resource availability. In resource-rich environments, dispersive configurations worked better in reducing vector population, and stations at or around houses worked better in preventing biting and parasite transmission. In resource-poor environments, all configurations worked similarly. Optimum numbers of bait stations should be adjusted according to seasonality when resource availability changes.

## Background

Indoor residual spraying (IRS) and insecticide-treated nets (ITNs)/long-lasting insecticidal nets (LLINs) have been widely used as malaria vector control tools and have achieved significant reduction of malaria transmission globally [1–5]. However, the increased use of pyrethroids for the treatment of nets has resulted in increased pyrethroid resistance in anopheline mosquitoes, reducing the efficacy of both IRS and ITNs/LLINs [6–8]. The use of these two indoor tools has also caused behavioural changes in anopheline mosquitoes in multiple locations over the world: the indoor host-seeking behaviour has shifted to a more exophilic (outdoors) behaviour [9–12]. It is questionable whether IRS and ITNs/LLINs alone will achieve malaria elimination [13].

Residual malaria transmission [14] has been consistently demonstrated even in areas with good coverage of IRS and LLINs; the World Health Organization has declared an urgent need for new vector control tools, one type of which is sugar baits [15]. Attractive toxic sugar bait (ATSB) is a new method that uses attractive plant substances in the bait; contact with the sugar elicits a lethal effect on the mosquito after feeding on the sugar and toxin mixture [16–18]. Several field trials have proved the effectiveness of ATSB in controlling anopheline mosquitoes [16–20]. In addition, the risk of developing resistance is low since several different oral toxins can be used with ATSB, and it could be a promising simple and low-cost tool to supplement IRS and ITNs/LLINs combating ongoing residual malaria transmission [19]. ATSB can be either sprayed on vegetation or used in bait stations, can be applied outdoors or indoors [21], and can be applied in different configurations [16–20, 22, 23]. Accordingly, it is necessary to determine the number of ATSB stations and configuration of these bait stations needed for optimal malaria control in a village. ATSB has been effective even in sugar-rich environments [18], where natural sugar sources compete with ATSB, but such variations in environmental resource availability impact the survival and human-biting behaviour of *Anopheles gambiae* [24], and may alter the selection of optimum distribution and frequency of ATSB applications. While several field studies have been performed to evaluate the effectiveness of ATSB, all of these, except for one that also used vector capacity as an outcome [18], have been more focused on the decrease of vector abundance as the outcome [16, 17, 19, 25]. Evaluating the impact of ATSB on malaria transmission is a step forward toward the assessment of the method.

While field studies and community trials may be the best methods for answering these questions, they are expensive and time consuming. In addition, it can be very difficult to identify several villages that are comparable in both environmental structure and human demographics, such that sufficient controls and replicates are available. Using the same village through several periods of time to compare different ATSB configurations is also problematic, because the trials have to be carried sequentially, and additional washout periods between trials are needed to control the carryover effects. In addition, seasonality can affect the comparability of trials. It is therefore reasonable to carry out this spatial individual-based modelling (IBM) study simulating several comparable environments to evaluate the impact of different ATSB configurations on malaria transmission, and it can be useful in guiding experimental field designs. Recently, a spatial IBM was developed to simulate the interactions of *An. gambiae* mosquitoes and their environment, such as sugar feeding, blood feeding, resting, and oviposition and the study demonstrated a great impact of environmental sugar sources and outdoor resting sites on survival of *An. gambiae* and malaria parasite transmission [24]. By adding a few new features regarding ATSB, such as the interaction between mosquitoes and ATSB, the model can be used to simulate and evaluate the outcome of ATSB applications in different densities and configurations particularly in habitats that are optimally or marginally suitable for *An. gambiae*.

The objective of this study is to determine the optimum configuration and number of ATSB stations for *An. gambiae* and malaria control in resource-rich and resource-poor environments. Employing the modified model, different ATSB configurations were simulated and their impacts on the survival of *An. gambiae* and malaria transmission were evaluated and compared. Entomological inoculation rate (EIR), the rate at which people are bitten by infectious mosquitoes, has a log-linear relationship with malaria prevalence [26] and provides a direct measure of mosquito-to-human malaria parasite transmission intensity [27–29].

## Methods

### Model design

The basic model design was described in detail in Zhu et al. [24] according to ODD (overview, design concepts, and details) protocol on IBMs [30, 31]. With a few adjustments, this model was used for the purpose of this study. JAVA 7 (Oracle Co, Redwood, CA, USA) and Mason package v17 [32] were used to develop this model.

For the evaluations, the size of the hypothetical simulated village was 600 × 600 m in the continuous space, and 25 houses were randomly located in a 100 × 100 m area in the centre of the village. Two environmental resource availability levels were simulated: in resource-rich environment, 50 natural sugar sources, outdoor resting sites, and larval habitats were randomly located in the whole village; whereas in resource-poor environment, 25 of each were randomly located. One-hundred humans were living in the village, and they moved around in the daytime and went back and stayed in their homes at night. Protection by LLINs or IRS was not simulated.

One-thousand male *An. gambiae* mosquitoes and 1000 female *An. gambiae* mosquitoes were simulated at the beginning, with age, location and gravid status randomly assigned to them. The maximum lifespan of *An. gambiae* was 10 days for male and 30 days for female [33–35]. If the mosquitoes could not find sugar or blood meals at night and remained hungry by morning, they would die of starvation. They could also die from reaching the life spans or feeding on ATSB. At night, male *An. gambiae* could sugar feed, rest and fly around, whereas female *An. gambiae* could also blood feed and oviposit.

In the model, males, if they were hungry, would begin to seek sugar sources; if they were fed, then they would seek resting sites to rest. Females need blood to develop eggs and need sugar to survive and fly [36]; females in the model, if they were in need of blood meal, would begin to seek a human host, but if they became too hungry before they could find a human, they would begin also to seek a sugar meal to provide energy for further activities. Because of the inhibition of host-seeking response in *An. gambiae* following blood feeding as recorded [37], in the model if the females were blood-fed and gravid but the eggs were not ready, they usually did not search for a blood meal; when they were hungry, they would seek sugar sources. But if they were very hungry, they would start to seek hosts as well, but sugar seeking would still be a priority. If they were fed and not hungry, they would begin to seek resting sites. If the females were ready to oviposit, they would seek a larval habitat, but if they became hungry, they would seek a sugar source first. Every blood meal would be kept track of in the model, and the probability of infection with malaria parasite for an uninfected female *An. gambiae* by biting a human was considered as 20 % [38–45]. After an extrinsic incubation period of 10 days [38, 46], the female would become infectious, and afterwards biting of a human would be counted as potential malaria infection and used to calculate EIR.

In the model, when seeking either sugar, blood, resting sites, or larval habitats, the mosquitoes were able to sense the targets in the circle around them, the radius of which was the maximum attractive distance of the target. If they could find one, they would fly toward the target directly, but if they could not, they would fly in a random direction for the current step, and begin seeking again in the next step. Two-thousand random movements, which represented 2000 m of flight, would result in an additional hunger level [47].

Density dependence was applied in the development in the larval stage. Eggs hatching rate was 70 % of eggs oviposited as recorded [48, 49]. Number of larvae in each larval habitat on each day was calculated as the number of hatched eggs on that day, plus number of larvae that had survived from the previous day, minus number of larvae that had pupated on that day [50]. Independent mortality ‘m’ (without impact of larvae density) of larvae was 0.1 per day [51]. The overall mortality of larvae on day t ‘M_t_’, considering the density-dependent mortality, was calculated as M_t_ = m × (1 + L_t_/K), where L_t_ is the number of larvae on day t, and K is the larval habitat capacity. K was assumed to be 300, deducting from the formula above, it means when the number of larvae in one habitat reached nine times of K (2700), the overall mortality would become one per day. Emerging rate from pupae to adult was 70 % of mature pupae [52]. Table 1 summarizes the parameters and assumptions used.

**Table 1:** Parameters and assumptions used in the model

Parameters/inputs	Values	References
Village size	600 × 600 m	Assumption
House distribution	100 × 100 m	Assumption
No. houses	25	Assumption
No. humans	100	Assumption
Initial no. male *An. gambiae*	1000	Assumption
Initial no. female *An. gambiae*	1000	Assumption
Human moving outdoors	07:00–20:00	Assumption
Active time of *An. gambiae*	19:00–05:00	Assumption
Max life span of male *An. gambiae*	10 days	[33–35]
Max life span of female *An. gambiae*	30 days	[33–35]
Hunger level threshold of sugar-seeking females switching to accepting blood	2	[37, 55–57] and assumption
Hunger level threshold of blood-seeking females switching to sugar-seeking	2	[37, 55–57] and assumption
No. random movements leading to an additional need for sugar meal	2000 steps	[47] and assumption
Extrinsic incubation period	10 days	[38, 46]
Minimum number of sugar meal of male *An. gambiae* per night	2	[53]
Minimum number of sugar/blood meal of female *An. gambiae* per night	1	[53]
Days needed to develop eggs after blood-feeding	2–3 days	[58]
Average size of egg batches	100	[59]
Attractive distance of sugar source	5 m	Unpublished data
Attractive distance of human	40 m	Unpublished data
Sensing distance of larval habitat site	5 m	Unpublished data
Sensing distance of resting site	5 m	Unpublished data
Duration of aquatic stage	12 days	[52, 60]
Larval habitat site capacity	300	Assumption
Egg hatch rate	0.7	[48, 49]
Independent mortality of larvae	0.1	[51]
Emerging rate of pupae	0.7	[52]

### Simulations

**Fig. 1:**
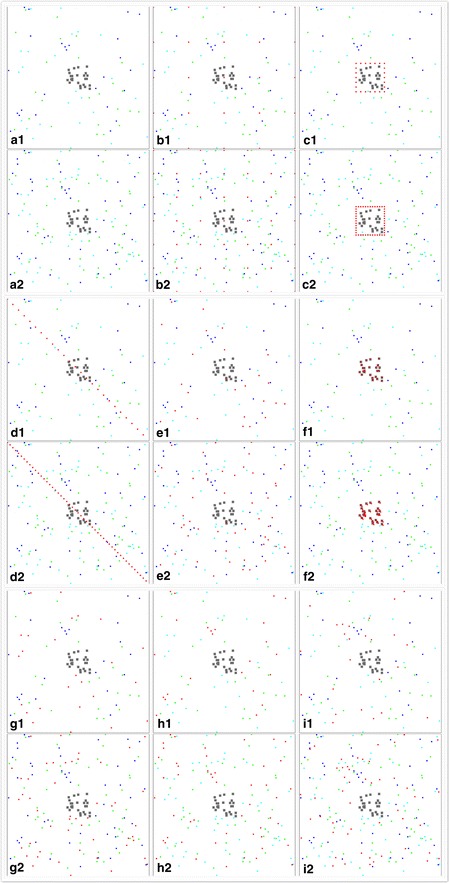
Configurations of ATSB stations in resource-rich and resource-poor environments. To be concise, the Figure contains only low-density (25) stations for resource-poor environments and high-density (50) stations for resource-rich environments. In each sub-figure, the *grey dots* represent houses, the *green dots* represent sugar sources, the *light blue* dots represent outdoor resting sites, the *dark blue dots* represent larval habitats, and the *red dots* represent ATSB stations. Sub-figures **a1**–**i1** are control, 5 × 5 grid design, house periphery design, transect design, stations at sugar sources, stations at houses, stations at resting sites, stations at larval habitats, and stations at random locations in resource-poor environments; sub-figures **a2**–**i2** are the same order of designs in resource-rich environments. In designs where stations were placed at resources (e.g., sugar sources), the *dots* representing the resources are hidden behind *red dots* and not shown. The series of n × n grid design are the same designs as **b1** and **b2**, except that the numbers in *each row* and *column* are 0, 2, 3, 4, 5, 6, 7, 8, 9

Eight types of ATSB station configurations and two density levels of each were simulated in resource-rich and resource-poor villages. The eight types of ATSB configurations were: n × n (7 × 7 or 5 × 5) stations placed in grid design over the entire area, 48 or 24 stations placed evenly at the periphery of the house area, 50 or 25 stations placed evenly in a transact across the village, 50 or 25 stations located within the natural sugar sources, 50 or 25 stations at houses, 50 or 25 stations within the resting sites, 50 or 25 stations at the larval habitats, and 50 or 25 stations randomly placed in the entire area. A scenario in which no ATSB station was placed out in the village was the configuration used as the control scenario. When two ATSB stations were put at each house, they were put 5 m apart in both the x and y horizontal directions. When two ATSB stations were put at each sugar source/resting site/larval habitat, they were put 1 m apart in both the x and y horizontal directions. After selecting an optimum configuration design, additional densities of ATSB stations placed in that design were simulated to determine the optimum number (a minimum number that is needed to achieve effective vector control that drives EIR to near zero) of stations needed. Figure 1 demonstrates the simulated map of the village and the locations of different objects. All ATSB stations were assumed to be working continuously for the whole period, because in reality, regular stations last for 1 month and bio-filmed stations work for 6 months, and they can be replaced easily.

**Fig. 2:**
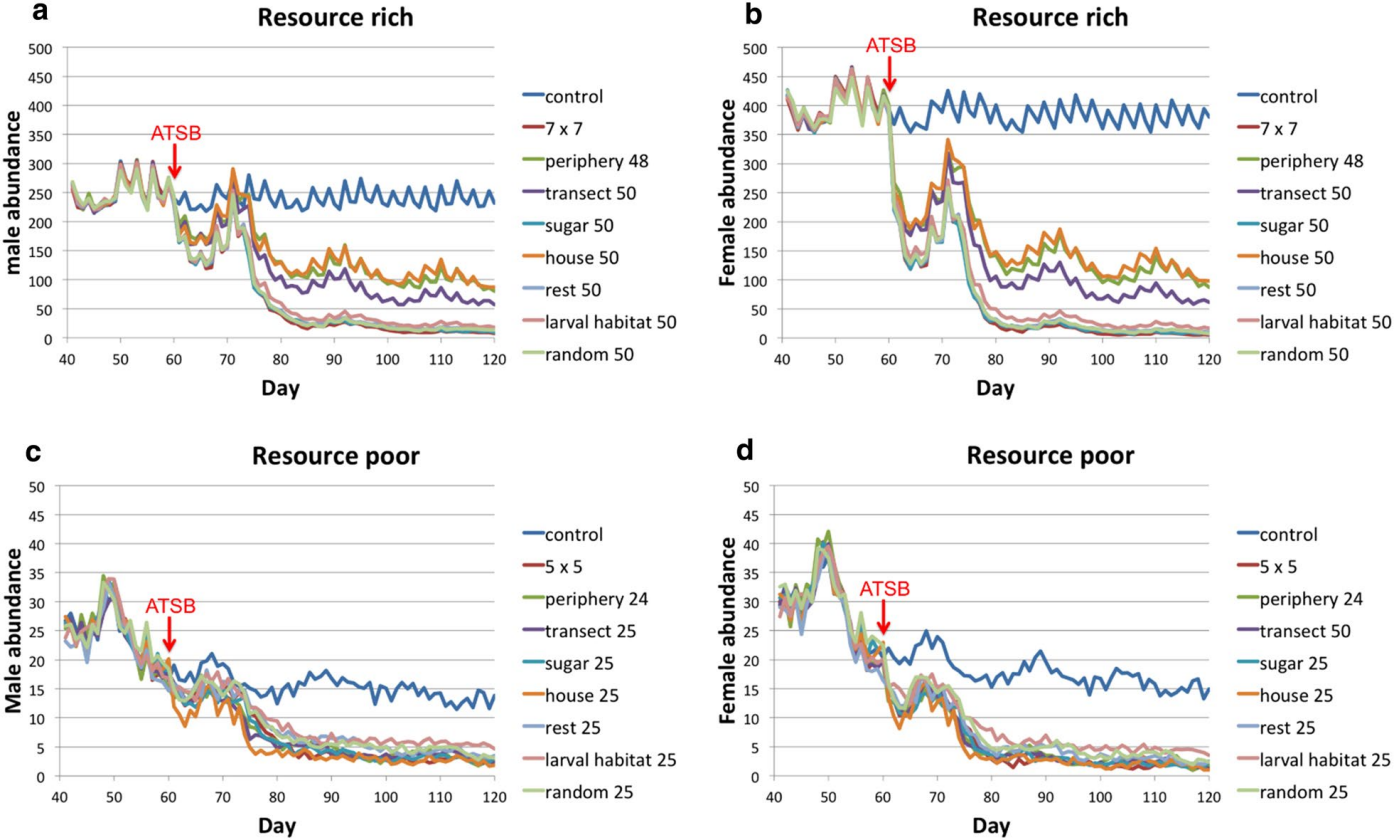
Average daily abundances for male and female *Anopheles gambiae* mosquitoes along time in simulations with different ATSB configurations. Sub-figures **a** and **b** are average daily abundances of male and female *An. gambiae* mosquitoes in resource-rich environments with different ATSB configurations. Sub-figures **c** and **d** are average daily abundances of male and female *An. gambiae* mosquitoes in resource-poor environments with different ATSB configurations. In each sub-figure, *x-axis* is the time in days; *y-axis* is the abundance (number of mosquitoes). Each *line* represents an ATSB configuration according to the legend on the *right*. The *red text* ‘ATSB’ and the *arrow below mark* the timing of ATSB treatment, which is at the beginning of day 61

Pilot simulations showed that in the control scenario, mosquito population equilibrated after day 40, with mild fluctuations. So, only the population dynamics after day 40 are shown in Fig. 2. ATSB stations were placed at the beginning of day 61, and the simulation continued to day 120, allowing time for the mosquito population to equilibrate again. Each scenario was simulated 50 times, each with a different pseudo-random initiator.

### Statistical analysis

Human biting rate (HBR) was defined as the total number of bites per day divided by the number of humans. Daily abundance was defined as the number of *An. gambiae* mosquitoes left at the end of each day before recruits of newly emerged adults. EIR was defined as the total number of infectious bites per day divided by the number of humans.

Only data after the population equilibrated (from day 101 to 120) were used for the comparison analysis. To provide the distributions of mosquito survival as a baseline information of the model, the means of daily survival rates for day 101–120 were calculated, and the average ages of all the mosquitoes at the time point of midnight of day 111 were calculated, both for the control scenario and the 7 × 7 design scenario in sugar-rich environments. The means of daily abundance, HBRs and EIR, and the percentages of decreases from control scenario were calculated. ANOVA and Tukey post hoc tests were used to compare outcomes of simulations of different ATSB configurations. SAS 9.3 (SAS Institute, Inc., Cary, NC, USA) was used for the analyses.

## Results

Table 2 shows the distribution of *An. gambiae* survival of ATSB-treated and untreated scenarios. ATSB treatment had greater impact on age composition in females than in males. At the time point of age recording (midnight of day 111), there was no female beyond extrinsic incubation period (EIP) left. ATSB treatment also substantially reduced daily survival rates in both males and females.

**Table 2:** Survival distribution of *Anopheles gambiae* in ATSB-treated and control environments

Sex	Treatment	Age					Daily survival rate	
		Mean	SD	Median	Min	Max	Mean	SD
Female	Control	4.2	4.6	2.2	0.2	29.2	0.81	0.05
	7 × 7 ATSB	1.4	1.7	0.2	0.2	9.2	0.32	0.39
Male	Control	2.3	2.4	1.2	0.2	9.2	0.72	0.05
	7 × 7 ATSB	2.2	2.4	1.2	0.2	9.2	0.41	0.43

Figure 2 shows the daily changes of male and female *An. gambiae* abundance with different ATSB configurations during the two-month period. To make the graphs more concise, only high densities of ATSB stations placed in resource-rich environments and low densities of ATSB stations placed in resource-poor environments are shown.

In resource-rich environments, male populations equilibrated at about 240 mosquitoes, and female populations equilibrated at about 380 mosquitoes. After the placement of ATSB stations at day 61, male and female populations in all ATSB-treated scenarios began to drop. Population sizes included adults newly emerged from pupae, which were resting for the first night and, therefore, did not approach the ATSB stations. Mosquitoes at this stage, that developed from eggs laid before ATSB placement caused a slight increase in the populations. However, with the beginning of ATSB effect on the rate of reproduction, the populations crashed again. Then both male and female population sizes equilibrated at about 60 (from about ten to 110 for males and from ten to 120 for females in different configurations). The optimum configurations to control mosquito populations were in the 7 × 7 grid design, 50 stations either at sugar sources, resting sites, larval habitat sites, or random locations. Stations placed at periphery of house area, at houses, or along a transect were less effective.

In resource-poor environments, the equilibrated number of males was about 14, and females equilibrated at about 16. After the placement of ATSB stations at day 61, all populations except the controls began to drop. A small increase in populations was also observed due to the delayed effect of pre-imaginal stages and thus on mosquito emergence. The equilibration level of both male and female populations was reduced to around or below five. Stations at houses and 5 × 5 grid design were most effective, and the differences between different ATSB configurations were negligible.

Table 3 shows the daily mean of mosquito abundance, HBR and EIR of scenarios with different ATSB configurations. In resource-rich environments, compared to the outcomes not altered by ATSB treatment in the control scenario, male abundance was reduced by 95.80 %, female abundance was reduced by 98.22 %, HBR were reduced by 99.52 %, and EIR was reduced by 99.99 %, with the optimum ATSB station configuration. In resource-poor environments, compared to the outcomes not altered by ATSB treatment in the control scenario, male abundance was reduced by 82.45 %, female abundance was reduced by 91.80 %, HBR were reduced by 98.15 %, and EIR was reduced by 100 %, with the optimum ATSB station configuration. The decreases in abundance, HBR and EIR were all significant in both resource-rich and resource-poor environments (*P* < 0.0001).

**Table 3:** Comparison of daily mean of abundance, human biting rate (HBR) and entomological inoculation rate (EIR) between different ATSB configurations and control

Environment	ATSB configuration	Male abundance		Female abundance		HBR		EIR	
		Means	% decrease	Means	% decrease	Means	% decrease	Means	% decrease
Resource rich	Control	239.375	0.00	380.781	0.00	6.69513	0.00	0.50085	0.00
	7 × 7	10.06	95.80	6.791	98.22	0.03203	99.52	0.00007	99.99
	5 × 5	31.285	86.93	28.168	92.60	0.18999	97.16	0.00088	99.82
	Periphery 48	96.289	59.77	108.078	71.62	0.66992	89.99	0.00152	99.70
	Periphery 24	99.447	58.46	111.905	70.61	0.69635	89.60	0.00176	99.65
	Transect 50	66.742	72.12	71.676	81.18	0.50966	92.39	0.00101	99.80
	Transect 25	70.795	70.43	75.996	80.04	0.56473	91.57	0.00089	99.82
	Sugar 50	12.723	94.68	9.375	97.54	0.04629	99.31	0.00011	99.98
	Sugar 25	29.25	87.78	26.644	93.00	0.17223	97.43	0.00042	99.92
	House 50	103.22	56.88	118.499	68.88	0.79739	88.09	0.00157	99.69
	House 25	104.922	56.17	121.827	68.01	0.83236	87.57	0.00185	99.63
	Rest 50	16.056	93.29	12.249	96.78	0.0697	98.96	0.00029	99.94
	Rest 25	36.883	84.59	35.806	90.60	0.27758	95.85	0.00198	99.60
	Larval 50	22.01	90.81	20.23	94.69	0.15867	97.63	0.0018	99.64
	Larval 25	49.956	79.13	53.629	85.92	0.51431	92.32	0.00763	98.48
	Random 50	14.545	93.92	11.493	96.98	0.07178	98.93	0.00009	99.98
	Random 25	42.344	82.31	43.48	88.58	0.37372	94.42	0.00643	98.72
	F	94.61		185.46		474.6		42.86	
	*P*	<0.0001		<0.0001		<0.0001		<0.0001	
Resource poor	Control	13.716	0.00	15.748	0.00	0.32559	0.00	0.01364	0.00
	7 × 7	2.544	81.45	1.292	91.80	0.00603	98.15	0.00003	99.78
	5 × 5	2.873	79.05	1.65	89.52	0.01071	96.71	0.00003	99.78
	Periphery 48	2.809	79.52	1.974	87.47	0.00898	97.24	0.00015	98.90
	Periphery 24	3.341	75.64	2.371	84.94	0.0113	96.53	0.00006	99.56
	Transect 50	2.601	81.04	1.765	88.79	0.0091	97.21	0.00012	99.12
	Transect 25	3.364	75.47	2.208	85.98	0.0123	96.22	0.0001	99.27
	Sugar 50	3.183	76.79	1.985	87.40	0.01208	96.29	0.00006	99.56
	Sugar 25	3.013	78.03	1.985	87.40	0.01162	96.43	0.00006	99.56
	House 50	2.407	82.45	1.957	87.57	0.00985	96.97	0.00032	97.65
	House 25	2.491	81.84	1.863	88.17	0.01057	96.75	0.00016	98.83
	Rest 50	3.316	75.82	2.215	85.93	0.01626	95.01	0.00009	99.34
	Rest 25	3.967	71.08	2.8	82.22	0.02411	92.59	0	100.00
	Larval 50	5.133	62.58	4.245	73.04	0.04778	85.33	0.0014	89.74
	Larval 25	5.609	59.11	4.48	71.55	0.04869	85.05	0.00099	92.74
	Random 50	2.745	79.99	1.873	88.11	0.01463	95.51	0.0001	99.27
	Random 25	4.234	69.13	3.316	78.94	0.03447	89.41	0.00004	99.71
	F	4.17		9.46		19.2		6.46	
	*P*	<0.0001		<0.0001		<0.0001		<0.0001	

Results of post hoc analysis showed that the differences of abundances, HBRs and EIRs between control and all the other ATSB configurations were significant (*P* < 0.0001). In resource-rich environments, control of female *An. gambiae* population was most effective with n × n grid design, designs of bait stations at sugar sources, resting sites, larval habitats, and random locations (*P* < 0.0001). There was no significant difference between the results within these configuration patterns (*P* > 0.05). Except for bait stations near larval habitats (P = 0.0322), there were no significant differences in the effects of high and low concentrations of ATSB stations on female abundance (*P* > 0.05). The estimated reduction of HBRs was similar to the results of female abundance. However, there was no significant difference of EIR reduction between all ATSB configurations (*P* > 0.05). In resource-poor environments, there was no significant difference in female abundance, HBRs and EIR between all ATSB configuration designs (*P* > 0.05).

**Fig. 3:**
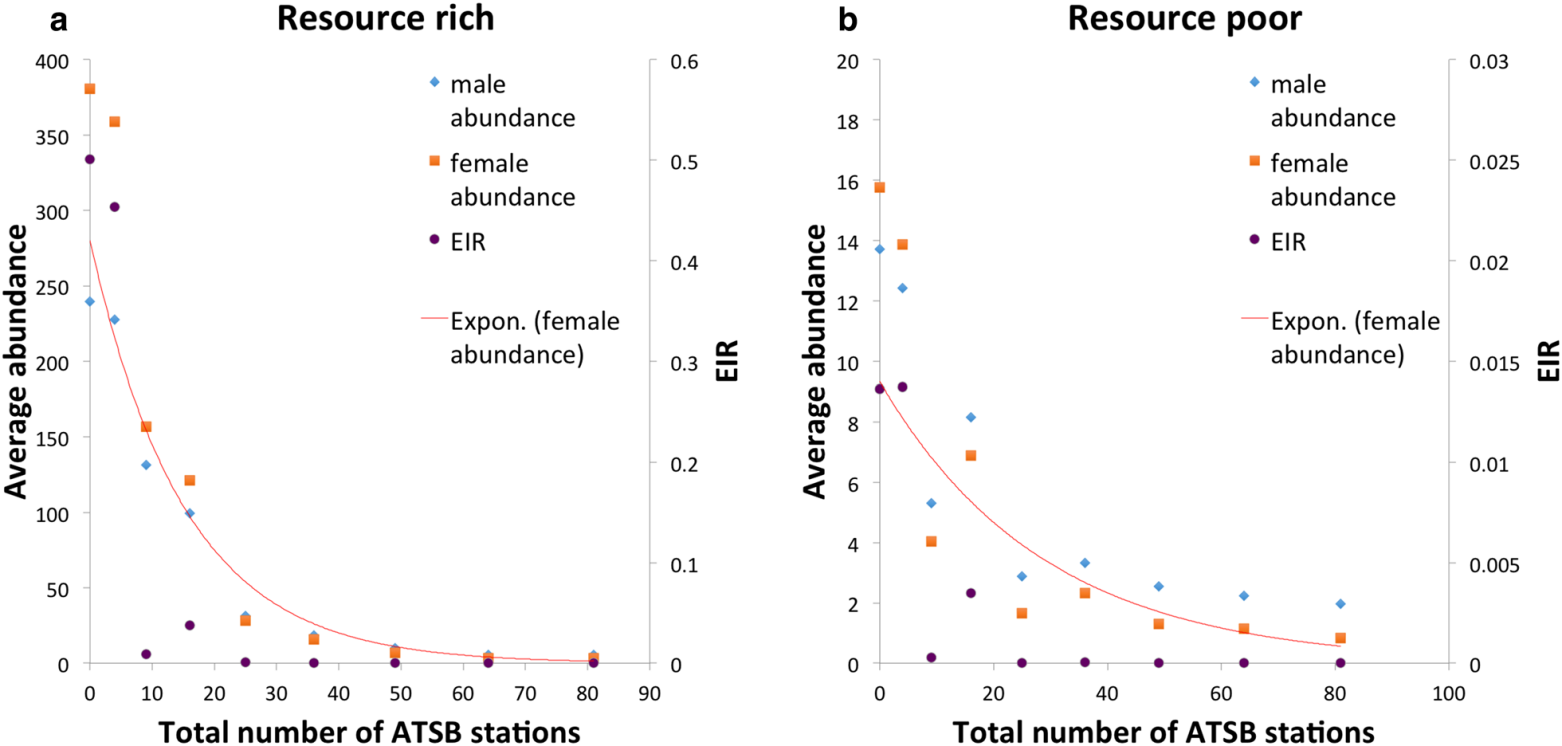
Average daily abundances for male and female *Anopheles gambiae* mosquitoes and EIR in simulations with different numbers of n × n grid design ATSB stations. This figure shows the average daily abundances for male and female *An. gambiae* mosquitoes and average EIRs calculated from the period from day 101 to 121 (after population equilibrium). Sub-figure **a** shows results in resource-rich environments, sub-figure **b** shows results in resource-poor environments. In each sub-figure, *x-axis* is the total number of ATSB stations; *y-axis* on the *left* is the abundance (number of mosquitoes) for the males and females; *y-axis* on the *right* is the EIR value. *Blue dots* represent male abundance, *orange dots* represent female abundance, and *purple dots* represent EIR. The 10 dots in each *colour*, from *left* to *right*, represent results with grid designs of 0, 2 × 2, 3 × 3, 4 × 4, 5 × 5, 6 × 6, 7 × 7, 8 × 8, 9 × 9

Based on the results that an n × n grid design was an optimum configuration in both resource-rich and resource-poor environments, it was selected for testing the impact of different numbers of ATSB station on vector control and EIR. Figure 3 shows the means of male abundance, female abundance, and EIR with 0, 2 × 2, 3 × 3, 4 × 4, 5 × 5, 6 × 6, 7 × 7, 8 × 8, and 9 × 9 ATSB stations in grid design in both resource-rich and resource-poor environments. An exponential trend line of female abundance was added for each plot. In resource-rich environments, mosquito population and EIR decreased rapidly when total number of stations increased from 0 to 25; after that, further increase of stations did not significantly improve the outcomes. In resource-poor environments, absolute numbers of mosquito populations and EIRs did not significantly decrease as the number of stations increased. However, the results demonstrate that after nine stations, the decrease was even slower.

## Discussion

Based on the spatial IBM, the study showed that ATSB application effectively reduced the density of *An. gambiae*, HBR and EIR in both resource-rich and resource-poor environments. Configurations of dispersed ATSB stations were significantly more effective for vector control in resource-rich environments; but in resource-poor environments, all configurations worked similarly. No significant difference in EIR reduction was found among all configurations in both resource-rich and resource-poor environments. Reduction of *An. gambiae* density and EIR increased with the increase in numbers of ATSB stations, but it reached a point at which further increase was ineffective.

Without ATSB treatment, female mosquitoes survived better than males in both resource-rich and resource-poor environments, but the difference in survival in resource-poor environments was negligible. However, after the ATSB treatment, male and female populations were reduced to very similar sizes, with the decrease in female numbers being greater than that of males. This finding was consistent with the observation for *Anophelesclaviger* reduction in an earlier field study in Israel, where average daily female catches per trap decreased from about 25 to under five, and average daily male catches per trap decreased from about 15 to under five [16]. The trend of population dynamics was also similar to the field trial in Mali showing that daily female catches decreased from approximately 180 to 25, and male catches decreased from approximately 90 to ten, where ATSB spray was used around larval habitats, and *An. gambiae* were collected by CDC light traps [17]. Similar population reduction trends were also observed in field trial of *Anopheles sergentii* [18]. The observed longer survival of females in untreated environments increases the risk of malaria transmission. It is, therefore, important to note that ATSB treatment effects on females are even stronger than on males.

In both resource-rich and resource-poor environments and with all ATSB configurations, percentages of reduction in HBRs were always greater than percentages of reduction in female populations, and percentages of reduction in EIRs were also always greater than percentages of reduction in HBRs. In other words, ATSB stations are more effective against females that are more likely to bite a human host, and females that carry malaria parasites. When comparing the increases from percentage reduction of female abundance to percentage reduction of EIR between different configuration designs, the intensity of increase was greatest in two configuration designs: bait stations at houses (e.g., percentage reduction increased from female abundance reduction of 68.88 % to EIR reduction of 99.69 % in resource-rich environments with 50 stations) and bait stations at the periphery of the house area (e.g., percentage reduction increased from female abundance reduction of 71.62 % to EIR reduction of 99.70 % in resource-rich environments with 50 stations). Although these two designs are not as good at reducing vector population, these results demonstrate that they have a better ability to target human-biting and parasite-transmitting mosquitoes. This can be explained by the results shown in Table 2: ATSB treatment killed most of the older mosquitoes and lowered the average age of the whole population. As the malaria parasite undergoes an EIP in the mosquito to become infectious, a female *An. gambiae* has to live longer than the EIP to transmit the parasite. During this time period the mosquito will need several sugar meals [36, 53], increasing the probability of the mosquito becoming attracted to and killed by ATSB before it becomes infectious. This is consistent with findings from a field study conducted in three oases in Sahara-Arabian phyto-geographical zone that ATSB treatment reduced the proportion of older more epidemiologically dangerous mosquitoes [18].

ATSB treatment is effective in both resource-rich and -poor environments. This finding is supported by the results of a previous field study concluding that ATSB decimate populations of *Anopheles* mosquitoes regardless of the local availability of sugar sources [18]. In resource-rich environments the mosquito population is higher than in resource-poor environments but the impact of ATSB application causes a similarly low population density in the treated areas whether the environment offers optimal conditions for mosquitoes or not. Thus, the difference of the treated areas from their respective controls is caused by differences in population size in the controls, which are not accompanied by a parallel variation in the treated areas. In other words, regardless of the availability of environmental resources and the initial similarity of the population size, ATSB treatment can reduce the population to a similar very low level. Moreover, with optimum ATSB station configuration, EIR can be reduced to below 0.0001 in both environments. Achieving this low level of EIR is important for malaria elimination strategies [28].

In resource-rich environments, configurations of dispersed ATSB stations were more effective in reducing mosquito populations than concentrated application configurations. However, EIR can be reduced by more than 99 % with 50 stations placed in any configuration. Placing the stations at the randomly distributed sugar sources, resting sites or larval habitats did not have any extra benefit over randomly placed stations at other locations. Placing stations at sugar sources, resting sites or larval habitats is not recommended. Additional benefits of this observation are the lower effort and expertise that are required to place bait stations in exactly identified locations that suit the mosquito biology. In resource-poor environments, all configurations of ATSB stations had similar effect as indicated by the lack of statistically significant difference. However, if fewer than 25 stations are used in the field in a resource-poor environment, as the one simulated in this study, the difference of impact between different configurations may become visible, and there might be an advantage of one configuration over others.

Use of bait stations at all houses in villages should be considered in field trials. Although placement of ATSB stations at houses was not the most effective configuration in resource-rich environments, it reduced the EIR by over 99 %. Operationally, placing ATSB stations at houses may be the most feasible and least expensive strategy. Location of ATSB stations at houses saves the labour of having to cover the entire village and periphery area. In addition, the ATSB stations at houses are more protected against damage and there is less need for replacement. Based on the current findings of this study, it is also likely that ATSB stations placed at each house to directly target human biting and parasite transmission may be most effective when houses are scattered rather than highly concentrated.

Placing an additional ATSB station at each house did not significantly increase mosquito mortality or reduce EIR. This finding was related to the assumption in the model that each ATSB could attract mosquitoes from all directions, so two stations close to each other did not work more effectively than a single station. In many areas in Africa, houses have a gap between the roof and the walls for ventilation, which accords with the assumption in this model that the odour of ATSB can distribute across the house. However, in other areas, where there are no gaps under house roofs, an additional ATSB station on the other side may be beneficial to optimize ATSB attraction.

A series of increasing numbers of ATSB stations in n × n grid design was tested to further explore the selection of optimum number of stations. For resource-rich environments simulated in this study (50 of each type of resources), increases in effectiveness were minor after the number of stations reached 25. For resource-poor environments simulated in this study (25 of each type), even ten stations can achieve satisfactory results; further increase of stations did not alter the outcome significantly. Therefore, using additional stations beyond the optimum number is not recommended. Seasonality was not simulated in this model; however, as seasons change, resource availability in the same village can change. The optimum number of stations should be adjusted according to the changes to ensure effectiveness. The highest number of ATSB stations are needed in rainy seasons when there is abundant sugar sources and resting sites available.

Extinction is not an issue in this study. Although a few repetitions ended up with zero mosquitoes at the end, as the average abundances over the last 20 days were calculated, it reduced the proportion of repetitions with the outcome of extinction. In addition, extinction only happened in the control/untreated scenario in resource-poor environments or scenarios with ATSB treatment, so it is a result of the given condition. Because of the stochasticity in the simulations, some repetitions had lower abundances, including extinction, and others had higher abundances; it evened out and the mean of the outcome values from the 50 repetitions was presented. Also, as the ‘highs’ and ‘lows’ happened randomly/equally in both the control and the treatment scenarios, it would not affect the comparison results.

Although the model simulated a hypothetical village in Africa, it can be generalized to other types of communities that need vector-borne disease control, with a few adjustments of parameters and modelling assumptions. There are some simplifications in the model that could influence the results. First, in the model, female *An. gambiae* had a constant rate of 20 % of becoming infected with malaria after biting a human. This was assumed to avoid the complexity in human malaria infections such as immunity. However, as EIR decreases, malaria prevalence could decrease too. So the assumption of a constant rate could result in an under-estimation of the impact of ATSB applications. Second, humans did not kill mosquitoes in the model, and IRS and LLINs were not simulated. This could result in an over-estimation of the abundance and EIR in all scenarios. An analysis of the synergistic impact of ATSB with LLINs and IRS on mosquito density is already modelled by Marshall et al. [54]. Third, successful mating was assumed in all females, so the decrease in male populations did not interact with or show any effect on female outcomes. However, if mating behaviour was incorporated in the model, the decrease in male populations may cause females to have to fly longer for successful mating, resulting in an increased need of energy sources. In addition, those females not mated will not produce eggs, which might further reduce the whole mosquito population. Thus, the impact of ATSB could have been under-estimated.

## Conclusions

In this model, application of ATSB stations significantly reduces *An. gambiae* abundance, HBR, and EIR in both resource-rich and resource-poor environments. All configurations of ATSB stations led to significant reduction of EIR to near zero, demonstrating a promising strategy for malaria elimination. In resource-rich environments, configurations of ATSB stations dispersed over the whole village achieved better control of mosquito vectors; among the dispersed ATSB station configurations, both the n × n grid design and the random location design are suggested. Stations at or around houses are less efficient in reducing vector population, but they work better in preventing biting and parasite transmission. As all ATSB station arrangements are similarly effective in resource-poor environments, any can be used. Modelling indicates one bait station at each house is an effective and feasible application strategy but in the field if it does not achieve satisfactory vector reduction, other dispersed configurations can be combined in parallel. Optimum number of stations should be adjusted according to seasonal changes in environmental resources, with highest numbers of bait stations placed during and after rainy seasons when there is an abundant of microhabitats where mosquitoes sugar feed and rest outdoors.
